# Mechanotransduction as a therapeutic target for brain tumours

**DOI:** 10.1016/j.ebiom.2025.105808

**Published:** 2025-06-16

**Authors:** Lauren Gomes, Carlos Pardo-Pastor, Jody Rosenblatt, Antonios N. Pouliopoulos

**Affiliations:** a School of Biomedical Engineering & Imaging Sciences, King's College London, London, United Kingdom; b Randall Centre for Cell & Molecular Biophysics, School of Basic & Medical Sciences, Faculty of Life Sciences & Medicine, King's College London, London, United Kingdom; c School of Cancer & Pharmaceutical Sciences, Faculty of Life Sciences & Medicine, King's College London, London, United Kingdom; d The Francis Crick Institute, London, United Kingdom

**Keywords:** Mechanotransduction, Brain tumours, Mechanosensitive ion channels, Piezo, Focused ultrasound

## Abstract

Despite decades of research, treatment options for many paediatric and adult brain tumours remain inadequate. Mechanotransduction, a process by which cells convert mechanical cues into biochemical signals, resulting in the activation of signalling cascades, is crucial in the progression of aggressive brain tumours such as glioblastoma (GBM). In GBM, a stiffened extracellular matrix accompanies the aberrant expression of mechanosensitive ion channels, including Piezo and transient receptor potential (TRP) channels, impacting brain tumour progression and therapeutic response. Thus, targeting these ion channels and associated signalling pathways may provide effective adjuvant therapy. Focused ultrasound (FUS) is an emerging technology being explored in diagnostic and therapeutic applications within oncology and has the potential to non-invasively modulate mechanosensitive pathways. Here, we discuss recent findings, highlighting how mechanobiology is altered in brain tumours, the potential of mechanosensitive ion channels as therapeutic targets and perspectives on using FUS to exploit aberrant brain tumour mechanobiology to provide non-invasive adjuvant therapy. At the intersection of cancer cell biology and biomedical engineering, this review offers a perspective on leveraging mechanotransduction for therapeutic advances in brain tumours.

**Search strategy and selection criteria**

To identify relevant literature for this review, we conducted a comprehensive search using OVID MEDLINE and PubMed databases. The search strategy employed combinations of keywords including “Mechanotransduction”, “Mechanobiology”, “Brain tumours”, “Glioblastoma”, “Diffuse midline glioma”, “Extracellular matrix”, “Mechanosensitive ion channels”, “Piezo”, “TRP”, and “Focused Ultrasound”. The search was restricted to peer-reviewed original research articles and review papers published in English from 1990 to 2025.

Following the initial search, the titles and abstracts of identified articles were screened for relevance. Full-text articles were then reviewed to assess their suitability for inclusion based on the following criteria:

1. Relevance: Studies were considered if they were directly related to mechanotransduction in cancer, with particular attention to brain tumours, and/or investigated mechanosensitive ion channels, their associated signalling pathways, and therapeutic strategies that modulate these mechanisms including the use of focused ultrasound.
2. Scientific quality: Only studies with clearly described methodologies, robust data, and coherent conclusions were considered.
3. Contribution to the field: Priority was given to studies that presented novel insights or offered comprehensive syntheses of existing knowledge.

Additionally, reference lists of the included articles were manually reviewed to identify further studies that met the above criteria.

## Introduction

Glioblastoma (GBM), a grade IV astrocytoma, is the most aggressive primary malignant adult brain tumour.^1^ Standard-of-care treatment includes maximal surgical resection, radiotherapy, and administration of the oral chemotherapeutic agent temozolomide (TMZ).^2^ Despite decades of research, the median length of survival post-diagnosis remains at 14.6 months.^2^^,^^3^ There is a lack of consensus on the precursor cell lineage of GBM. However, GBM tumours have been proposed to arise from the malignant transformation of neural stem cells, oligodendrocyte precursor cells, or astrocytes.^4^ GBMs are characteristically highly confined solid tumours with inter- and intra-tumour heterogeneity.^5^ Tumour cells are highly invasive and migrate into surrounding healthy tissue and parenchyma distant from the primary tumour site. This, combined with indistinct tumour margins, impedes efficient maximal surgical resection, contributing to the high incidence of GBM recurrence.^6^, ^7^, ^8^

Another aggressive brain tumour is paediatric diffuse midline glioma (DMG), formerly known as diffuse intrinsic pontine glioma (DIPG). DMG may result from malignant differentiation of neural stem cells of the oligodendroglia lineage since they resemble oligodendrocyte precursor cells as the cells of origin.^9^, ^10^, ^11^ DMG is an incurable brain tumour, found predominantly in midline brain structures, including the thalamus and brain stem.^12^ Due to the infiltrative nature of DMG, these tumours can spread to other brain regions and the spinal cord. The proximity of the tumour to critical brain regions, its diffuse nature, resistance to chemotherapy drugs, and insensitivity to radiotherapy render it inoperable, which leads to standard treatment consisting of palliative radiotherapy and end-of-life care,^13^ with a median survival of 10.4 months from diagnosis.^14^

While GBM and DMG are aggressive and treatment-resistant, other tumours, including benign tumours such as craniopharyngiomas, also present significant surgical challenges due to their location in the skull base and proximity to critical structures, including the pituitary gland.^15^

In summary, poor prognosis and a lack of treatment options demand new therapeutic approaches to tackle brain tumours, including GBM and DMG. Focused ultrasound (FUS) is an emerging technology within oncology, and here we discuss its potential to non-invasively exploit aberrant mechanobiology in brain tumours. In this review, we aim to highlight recent findings on the mechanobiology of cancer cells and discuss the potential of mechanotherapy, particularly FUS, as an innovative treatment strategy to address this unmet medical need.

## Mechanotransduction

Living organisms are exposed to a range of forces, from macroscopic forces such as gravity and bulk anatomical movement to localised microscopic mechanical forces, including fluid shear stress and compressive forces. These diverse forces influence cellular behaviour, including tumour progression. During brain tumour growth, tumour tissue displaces or infiltrates neighbouring healthy tissue, leading to significant compression and deformation. Mechanical forces, including tumour-associated oedema and tumour growth-induced solid stress (i.e., tensile and compressive forces exerted on surrounding tissue), contribute to the compression and deformation of brain tissue by the tumour mass.^16^^,^^17^ These forces also impact the development and properties of the tumour itself. Brain tumours develop differently depending on mechanical cues and spatial restrictions imposed by the extracellular environment, highlighting the complex interaction between the tumour and surrounding tissue.^18^ In addition to solid and shear stresses, extracellular fluid viscosity can influence tumour cell behaviour. Elevated viscosity has been shown to promote cancer cell motility and dissemination via actin cytoskeletal reorganisation, NHE1-mediated cell swelling, and activation of ion channel TRPV4.^19^ While not yet studied in GBM, these findings underscore the broader significance of fluid mechanical properties in tumour progression and highlight a potential area for future investigation in brain tumour mechanobiology.

Mechanotransduction describes the cellular process by which cells sense and respond to mechanical stimuli.^20^ This process involves the conversion of mechanical cues into biochemical signals, which subsequently modulate cell signalling cascades and dictate cellular fate outcomes. Cells express a variety of proteins activated in response to various mechanical stimuli such as pressure, stretch, or shear flow.^20^ In this article, we focus on a group of transmembrane proteins activated by increases in membrane tension that are central to mechanotransduction: mechanosensitive ion channels. We refer readers to recent reviews about other mechanosensitive proteins, including integrins^21^^,^^22^ or cadherins,^23^^,^^24^ as well as reviews on other ion channels found to have altered expression in gliomas that will not be discussed here.^25^

Mechanosensitive ion channels allow cells to sense changes in their mechanical environment and adapt to them, playing an integral role in physiological processes.^26^^,^^27^ These ion channels are expressed by various cell types and are located throughout the body, serving numerous essential physiological functions such as detecting touch, facilitating muscle contractions, and sensing changes in cell volume and extracellular matrix (ECM) stiffness.^28^^,^^29^

Mechanical cues from the ECM, including pressure, stretch, or shear flow, which are detected by cells via mechanosensitive ion channels, control normal cell function. ECM remodelling is commonly observed in diseases, including cancer progression.^30^^,^^31^ The ECM is an organised 3-dimensional structural network surrounding cells with important architectural and functional roles in physiological tissue organisation and remodelling. Mechanosensing and mechanotransduction involve ECM constituents found extracellularly, including proteoglycans, collagens, elastins, fibronectins, and laminins.^32^ Integrins are transmembrane heterodimeric cell surface receptors that coordinate cell signalling, proliferation, and migration.^33^ The extracellular region of integrins interacts with ECM components, including fibronectin, and regulates cell adhesion and migration in both normal physiology and in tumorigenesis.^34^ Piezo1, a mechanosensitive ion channel, has been shown to play a key regulatory role in activating integrins through calcium and R-Ras signalling, providing evidence for Piezo1 as a modulator of integrin-mediated cell adhesion.^35^

## Brain tissue mechanics

Mechanical properties of brain tissue, such as stiffness, in both the healthy brain and under pathological conditions, have been widely studied using various techniques, including magnetic resonance elastography (MRE)^36^^,^^37^ and atomic force microscopy (AFM).^38^^,^^39^ Disease states can lead to aberrant tissue mechanics, such as an observed decrease in brain stiffness in diseases like Alzheimer's.^36^

Young's modulus is frequently used as a measure of compressive brain tissue stiffness, due to its ability to characterise a material's composition and structural properties. Young's modulus describes the amount of force required to deform an object and is a measure of a material's intrinsic ability to resist deformation, independent of its shape or size. It is expressed in units of force per area (N/m^2^) or Pascals (Pa), where a higher Young's modulus indicates a stiffer material.^40^ However, it is important to note that while stiffness and Young's modulus values are related, stiffness is a measure of an object's resistance to deformation and depends on both the material properties and its geometry. Within the brain, stiffness varies significantly across different regions and measurements of stiffness depend on the techniques used.^41^ Tissue stiffness is influenced by ECM deposition or degradation, ECM cross-linking, changes in microarchitecture, and cellular-level factors such as cytoskeletal remodelling.^20^

The composition and mechanics of the ECM are influenced by the interactions between the cells and ECM in a feedforward cycle.^20^ Local changes in ECM composition and stiffness will impact the surrounding cells and mechanical cues control cell function including proliferation, invasion, and migration in brain tumours.^30^

Viscoelasticity describes a property of materials, such as brain tissue, that exhibit both viscous and elastic characteristics. These materials undergo continuous and irreversible deformation under force, which leads to energy dissipation and stress relaxation and is a key measurement of interest in understanding brain tissue mechanics. Combined with stiffness, viscoelasticity is a crucial measurement in brain tissue mechanics because it provides key insights into how brain tissue responds to mechanical stress and is affected in disease states.^42^

## Mechanical hallmarks of cancer: mechanobiology in the brain tumour microenvironment

### ECM stiffness in GBM

AFM is used to quantify the stiffness of the ECM in brain tumours. In fresh-frozen human samples, GBM tumours exhibit increased stiffness compared to low-grade gliomas.^43^ Subsequent studies have also shown that stiffness determined by AFM in orthotopically xenografted, patient-derived GBM tumours is observed to be 10 ± 7 and 4 ± 3 times higher, at the core and edge regions, respectively, than non-cancerous peritumoral brain tissue.^44^ In contrast, MRE, a non-invasive technique that measures the mechanical properties of tissues in vivo, found GBM tumours and other gliomas to be softer than healthy brain tissue.^45^, ^46^, ^47^ Streitberger et al. (2014) showed, using MRE, that GBMs were on average softer (1.32 ± 0.26 kPa) compared to healthy brain tissue (1.54 ± 0.27 kPa), although some GBM tumours (5 out of 22) exhibited increased stiffness.^45^ Also using MRE, Pepin et al. (2018) found healthy brain tissue to be stiffer (3.3 kPa) than previously reported by Streitberger et al. Although gliomas were stiffer (2.2 kPa) than those reported by Streitberger et al., they were still softer than healthy brain parenchyma included in the same study (3.3 kPa).^46^ Additionally, other work reports that MRE observes intra- and intertumoral stiffness heterogeneity in gliomas.^47^ The inconsistencies between AFM and MRE findings regarding tumour stiffness are intriguing, with a potential explanation being the difference in the scales at which these techniques measure stiffness.^36^, ^37^, ^38^, ^39^ AFM measures the local stiffness of the ECM at the micro- to nanoscale, whereas MRE assesses the bulk mechanical properties of tissues at a macroscopic level. This discrepancy might reflect the heterogeneous nature of tumour tissue, where localised stiff regions (measured by AFM) coexist with softer bulk properties (measured by MRE). Understanding these differences is crucial for interpreting the biomechanical characteristics of gliomas.

In vitro studies demonstrate that microenvironmental stiffness regulates glioma cell proliferation, as growing glioma cells on stiff substrates promotes proliferation.^48^^,^^49^ Changes in the stiffness of the ECM, tumour tissue stiffness and cell-ECM interaction importantly regulate migration, invasion, and proliferation of GBM tumours via activation of mechanosensitive ion channels and downstream signalling pathways that support pro-tumour processes.^31^ Cells typically migrate toward regions of increasing stiffness, a process termed durotaxis. Glioblastoma cell lines U87-MG and T98G have been shown to exhibit strong positive durotaxis.^50^ However, there is also evidence that suggests that another glioma line, U-251-MG, instead exhibits negative durotaxis, migrating toward a less stiff environment.^51^ In this study, fewer U-251-MG cells were found in the softest regions, suggesting that at below optimal stiffness, cells migrated via positive durotaxis.^51^ These studies highlight the complexity of mechanosensitive behaviours in GBM progression.

The brain tumour microenvironment (TME) describes a complex network encompassing various cellular components in the region surrounding a tumour. In addition to cancer cells, the brain TME is composed of vasculature, endothelial cells, neurons, and glial cells such as astrocytes (which are the most abundant type of glial cells that provide structural, metabolic, and homoeostatic support in the central nervous system; CNS), as well as microglia (the resident immune cells of the brain).^52^ An altered ECM is a key component of the brain TME and provides structural support to the tumour.^31^ The functionality of the blood vessels and cellular networks within the brain TME plays a crucial role in tumour development, progression, and response to therapy.^53^ Due to the aggressive growth dynamics of GBM and the constrained spatial environment of the brain within the skull, confined tumour cells are exposed to elevated pressure exerted by the surrounding tissue as tumour growth progresses. The increase in pressure from the cancer growth causes remodelling of the ECM, which can include increased ECM and tumour stiffness and altered microarchitecture. These changes in mechanical properties directly influence cancer development and are mechanical hallmarks of cancer.^54^

Viscoelastic studies in a mouse model have also shown that in the implantation of the GBM stem cell line (G30), the viscoelastic modulus in the central tumour region was significantly reduced compared to healthy brain tissue.^55^ Moreover, others have reported that gradients in tissue viscoelasticity between tumours and healthy brain tissue^56^ may facilitate tumour invasiveness and targeting this could be a valuable avenue. This reduction in viscoelasticity may facilitate tumour invasiveness by enabling cells to migrate more easily through the brain's mechanically adaptive environment. Targeting viscoelastic properties, particularly stress relaxation, could offer a valuable therapeutic approach to limiting GBM progression.

## Mechanosensitive ion channels implicated in brain tumours: TRP and Piezo

### TRP ion channels

Transient receptor potential (TRP) channels are a superfamily of 28 cation permeable channels, with roles involved in a vast array of physiological and pathophysiological sensory processes including heat, cold, pain, stress, vision and taste.^57^ The 7 subfamilies that the TRP ion channels have been sub-classified into are TRPA (Ankyrin), TRPC (Canonical), TRPM (Melastatin), TRPML (Mucolipin), TRPN (NO-mechano-potential, NOMP), TRPP (Polycystin), TRPV (Vanilloid). These ion channels are expressed by multiple cell types and are found in various tissues including the brain; diverse stimuli activate them and are permeable to a range of cations including Ca^2+^, Mg^2+^, Na^+^, and K^+^. An excellent 2024 perspective highlights the lack of evidence supporting the role of TRP ion channels as primary detectors of mechanical stimuli.^58^ This review details that these ion channels may not directly sense and convert external mechanical stimuli into intracellular signals and act as primary mechanotransducers but instead respond to the activity of mechanoreceptors such as Piezo1 acting as ‘mechanoamplifiers’ within the mechanosignalling pathway.^58^ TRP channels have been shown to promote tumour progression by facilitating cancer cell invasion and metastasis through signalling pathways. Specifically, TRPC1, TRPC6, TRPM2, TRPM3, TRPM7, TRPM8, TRPV1, and TRPV2 channels are highly expressed in patients with glioblastoma.^59^ TRPM8 is particularly highly expressed (1850-fold change), with the next highest channel TRPC6 (137.5-fold change).^59^ Interestingly, in the patients who survived more than 12 months, the overexpression of TRP genes was significantly greater compared to the patients who survived less than 12 months.^59^ These results suggest that selected TRP channels are expressed more as the disease progresses and that these ion channels act as important mediators of the progression in patients with GBM and indicate a potentially beneficial role for these ion channels in terms of survival.^59^ However, it is important to note that the control for this study was only an n = 1, as brain tissue sampling is not clinically routine or ethical for healthy participants. Further evidence has been provided for the role of ion channel TRPM7 in GBM invasion, whereby inhibition or knockdown of TRPM7 reduces human glioma cell migration and invasion.^60^ A TRPM7 antagonist, waixenicin A, reduces cell viability, migration, and invasion in two in vitro GBM models (U-87 and U251 cells).^61^

### Piezo ion channels

The term “Piezo” is derived from the Greek word “piesi” referring to pressure. The *FAM38A* and *FAM38B* genes encode Piezo1 and Piezo2 ion channels, respectively. These are Ca^2+^ permeable mechanosensitive ion channels that can transduce a range of mechanical stimuli into biochemical signals and are emerging as key mediators of cancer progression.^26^^,^^62^ Functional Piezo channels are large trimers with each monomer comprising more than 2500 amino acids.^62^ Mechanical forces including shear stress, pressure, and membrane tension are shown to activate Piezo channels and induce the influx of cations including Ca^2+^, Mg^2+^, and Na^+^.^63^ Interestingly, Piezo1 knockdown in neural stem cells shifts differentiation towards increased astrocyte formation and decreased neuron formation, indicating the role of Piezo1 in neuronal-glial lineage specification.^64^ We refer readers to a recent review on the role of Piezo1 in the CNS.^65^

Numerous studies have highlighted that Piezo1 is highly expressed in brain samples from patients with high-grade gliomas compared to low-grade gliomas and normal brain tissue samples.^66^, ^67^, ^68^ Piezo1 expression levels increase proportionally to glioma grade, and in GBM, Piezo1 expression was shown to be upregulated (2.43-fold change) compared to normal brain tissue.^66^ Due to this overexpression, high-grade glioma cells are suggested to be mechanosensitive. Piezo1 expression correlates with glioma grade, with higher expression in more aggressive, high-grade tumours, suggesting its role in tumour progression and severity.^66^, ^67^, ^68^ Subsequently, Piezo1 has been proposed as a prognostic indicator and a potential therapeutic target in patients with GBM.^66^, ^67^, ^68^

Piezo2 is distinct from Piezo1 in structure with key differences in domains, including the cap, distal blade and beam domains, which influence its gating kinetics and mechanosensitivity.^62^^,^^69^ Piezo2 plays an important role in touch sensation and is expressed in subsets of somatosensory neurons^70^ and Merkel cells.^71^^,^^72^ The expression of Piezo2 in GBM and DMG has not been investigated. However, several studies have utilised Piezo2 knockdown models to investigate its role in brain tumour progression. One study revealed that tumour growth was suppressed in a mouse glioma model post-subcutaneous implantation of Piezo2 knockdown GL261 cancer cells.^73^ Furthermore, the same study demonstrated that Piezo2 is localised in the endothelial cells that line tumour blood vessels, termed tumour endothelial cells. Piezo2 knockdown decreased tumour vascular density, suggesting that Piezo2 has a key role in regulating tumour angiogenesis as well as tumour growth and is a potential anti-angiogenic therapeutic target.^73^ In another study, mouse medulloblastoma cells expressing Sox2^+^, a genetic marker of chemoresistance, were found to ensheath capillaries, thereby creating a blood-tumour barrier (BTB) that reduces therapeutic agent access to the tumour.^74^ Remarkably, when Piezo2 is knocked out in vivo, the BTB is impaired, suggesting that its integrity may depend on Piezo2 expression. In the same study, the Piezo2 knockout also reversed detrimental WNT/β-catenin signalling states, reduced cancer cell quiescence, and enhanced chemotherapy response, implying that inhibition of Piezo2 may address BTB integrity and tumour quiescence.^74^

Mechanobiological responses following Piezo2 activation have also been explored. Piezo2 has been proposed to be essential for brain metastatic cells from breast cancer (MDA-MB-231-BrM2) to sense and navigate their physical environment as they migrate through confined narrow pores. Μoreover, Piezo2 was essential for the synthesis and release of serpins, which shield invading cells from brain defence mechanisms mediated by plasminogen activator.^75^^,^^76^ Piezo2-dependent Ca^2+^ influx was shown to activate RhoA, a small GTPase protein that promotes the formation of actomyosin stress fibre (SFs) and focal adhesions (FAs), critical for mechanical force transmission, mechanotransduction, and cancer invasion.^75^ Others have also demonstrated the importance of SFs and FAs in force transmission and mechanotransduction.^77^ Given the poor prognosis of breast cancer brain metastases and the limited efficacy of current treatments,^78^ understanding the mechanobiology of metastatic cells could offer new therapeutic insights.

## Downstream signalling pathways involved in brain tumours

### YAP/TAZ

The yes-associated protein (YAP) and PDZ-binding motif (TAZ) are transcriptional co-activators and constituents of the Hippo signalling pathway. YAP/TAZ plays essential roles in cancer progression as a promoter of cell proliferation, migration, chemoresistance, and apoptosis evasion.^79^^,^^80^ YAP/TAZ is phosphorylated in the cytoplasm and once is dephosphorylated, the complex translocates from the cytoplasm to the nucleus where it binds to the transcriptional enhanced associate domain (TEAD), promoting cell proliferation.^81^^,^^82^ In parallel, mechanical signals like substrate stiffness, cell spreading, or nuclear flattening promote YAP/TAZ nuclear accumulation via Rho-dependent actomyosin contractility, independently of Hippo signalling.^83^^,^^84^

**Fig. 1:**
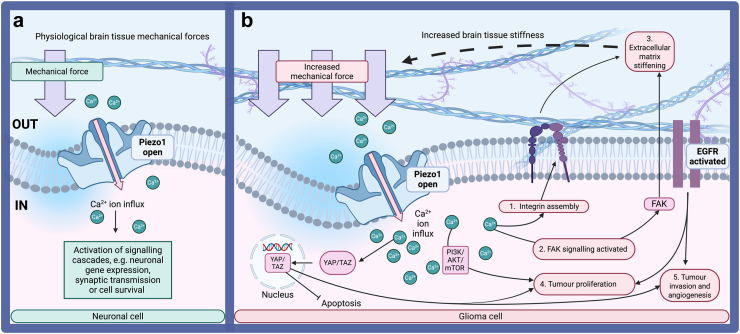
A proposed model of a potential Piezo1-dependent feedforward mechanism that potentiates extracellular matrix (ECM) stiffness and tumour progression. (a) Under physiological mechanical forces, Piezo1 expressed in neuronal cells transduces mechanical cues into biochemical signals, e.g., intracellular Ca^2+^ increases that influence normal physiological behaviour, such as synaptic transmission. (b) In contrast, during brain tumour growth, spatial constrictions by the surrounding tissue impose compressive mechanical forces on the expanding tumour mass. These mechanical stresses may repeatedly activate Piezo1, potentially leading to an overall intracellular increase of Ca^2+^ and other cations. This activates several signalling cascades, including focal adhesion kinase (FAK), phosphoinositide 3-kinase/protein kinase B/mammalian target of rapamycin (PI3K/AKT/mTOR), and Yes-associated protein/transcriptional co-activator with PDZ-binding motif (YAP/TAZ) pathways. Piezo1 activation also promotes integrin assembly and ECM remodelling, further enhancing tumour cell proliferation, survival, and invasiveness. Epidermal growth factor receptor (EGFR) activation via Piezo1 activation is associated with angiogenesis, proliferation, and invasion. ECM stiffening resulting from these processes may create a reinforcing cycle that contributes to glioma progression. Created with Biorender.com.

Piezo1 knockdown in human neural stem cells leads to the exclusion of YAP from the nucleus, suggesting that Piezo1 could regulate YAP localisation.^64^ The hierarchical relationship between Piezo1 activation and YAP/TAZ signalling has been explored in several cancer types, including cholangiocarcinoma (bile duct cancer)^85^ and cervical carcinoma cells (HeLa),^86^ where Piezo1 activation via its specific agonist Yoda 1 increases YAP translocation to the nucleus.^85^ Piezo1 has been suggested to be a transcription target of YAP/TAZ signalling and an important cell signalling pathway in mediating cell proliferation in oral squamous cell carcinoma.^87^ YAP/TAZ can inhibit apoptosis in cancer cells by interacting with TEAD transcription factors, promoting cancer progression (Fig. 1). Abnormal nuclear expression of YAP/TAZ is linked to poor cancer prognosis and chemotherapy resistance.^88^ Immunohistochemical analysis of normal healthy cortical brain tissue highlighted that YAP and TAZ expression is limited to vascular cells and not evident in neurons and glial cells. However, high nuclear expression of YAP was observed in 75% of GBM samples, and both YAP and TAZ are highly expressed in 61% of GMB samples.^89^

Overall, these findings highlight that YAP/TAZ signalling, promoted by mechanical signals via Piezo1, plays a crucial role in brain tumour progression by regulating key processes such as cell proliferation, survival, and resistance to chemotherapy.

### FAK

Focal adhesion kinase (FAK) is a non-receptor tyrosine kinase overexpressed in cancer cells.^90^ Piezo1 activation and subsequent calcium entry promote FAK signalling.^91^ Tumour stiffness and FAK phosphorylation (a marker of its activation) typically increase as tumours progress from the less stiff lower-grade gliomas to the stiffer GBM tumours.^43^ FAK mediates signalling from the ECM to the cytoplasm via several pathways, including the phosphatidylinositol 3-kinase (PI3K)/protein kinase B (AKT)/mechanistic target of rapamycin (mTOR) pathway that promotes cancer cell survival.^92^ FAK plays key roles in cell invasion, migration, and adhesion and mediates stiffening in GBM (Fig. 1). In vitro, FAK inhibitor (FAKi) defactinib has been found to cause senescence of GBM cells.^93^ Cells that enter senescence stop proliferating due to irreversible cell cycle arrest which can serve as a tumour suppressive function.^94^ In the same study, another FAKi (PF-573228) reduced GBM neurosphere growth and increased factors associated with cell senescence, such as elevated CDKN1B/p27 microRNA levels, suggesting that FAKi may arrest GBM cell proliferation. Subsequently, further clinical trials exploring FAK inhibition are ongoing (NCT05798507). Another small molecule FAKi, GSK2256098, is showing good tolerance in patients who have relapsed with GBM.^95^ However, larger multi-centre clinical trials are necessary to test the effectiveness of the drug.

### PI3K/AKT/mTOR

PI3K/AKT/mTOR signalling is involved in cancer progression by promoting the proliferation of glioma cells^82^^,^^96^ (Fig. 1). As gliomas have a notably aberrant PI3K/AKT/mTOR pathway, it is actively being pursued as a target for inhibitors. Alpelisib, a selective inhibitor of PI3K, effectively reduced both PI3K/AKT signalling and glioma spheroid growth.^97^ Additionally, Paxalisib, a PI3K/mTOR inhibitor, is in a phase II clinical trial as a monotherapy or in combination with ONC201 (NCT05009992) for DMG. In melanoma cancer cells, knockdown of Piezo1 gene expression inhibits the PI3K/AKT/mTOR pathway and Piezo1 activation via Yoda1 administration increases PI3K/AKT/mTOR protein levels, demonstrating the relationship between Piezo1 and PI3K/AKT/mTOR signalling.^98^

### EGFR mutations in GBM

The activation of the epidermal growth factor receptor (EGFR), a type of receptor tyrosine kinase that is overexpressed in GBM,^99^^,^^100^ results in the activation of multiple downstream signal transduction pathways related to cancer progression, including the PI3K/AKT/mTOR pathway.^101^ Moreover, up to 31% of GBM express constitutively active EGFRvIII, contributing to angiogenesis, proliferation, and invasion.^102^^,^^103^ Unlike other cancers, in glioma, EGFR mutations are in extracellular rather than intracellular domains, with up to 30% of GBM expressing constitutively active mutants (e.g., R108K, G598D, EGFRvIII).^102^^,^^103^ Constitutive activation of EGFR can contribute to angiogenesis, proliferation, and invasion and correlates with shorter survival.^102^^,^^103^ Remarkably, EGFRvIII is a deletion mutant lacking its ligand-binding domain, meaning mutations in EGFR can lead to continuous signalling even in the absence of EGF.^104^ GBM shows increased EGFR components and signalling in response to increased ECM stiffness.^48^ It has been reported that mechanical signals activate EGFR signalling via Piezo1. Importantly, EGFR ligand binding, tyrosine kinase activity, and tyrosine phosphorylation are not essential for this. Instead, this process relies on SRC- and p38-dependent serine phosphorylation, potentially explaining why GBM tumours often resist EGFR-targeted therapies, such as tyrosine kinase inhibitors.^86^

### Potential Piezo1-mediated feedback in GBM progression and ECM stiffening

The upregulation of Piezo1 in the progression, migration, and cell-death avoidance of GBM may act as a positive feedback cycle (Fig. 1). The fast-paced, confined growth of GBM means that brain cancer cells are under constant mechanical forces (including shear stress, compression, and tension) that activate Piezo1. In turn, Piezo1 activates FAK and integrin assembly, ECM remodelling, and YAP/TAZ and PI3K/AKT/mTOR signalling.^105^ This contributes to cell proliferation and migration whilst reducing apoptosis, resulting in tumour progression and ECM stiffening. This stiffened ECM further increases mechanical stress on GBM cells, perpetuating Piezo1 activation and sustaining a positive feedback loop. Due to this feedback cycle, ECM stiffening during GBM progression correlates with poor prognosis.^67^ It is important to add that sustained mechanical stimuli will activate other mechanosensitive components, such as TRP ion channels, and these may also be important mediators of tumour progression. Understanding these interconnected mechanotransduction mechanisms could provide new therapeutic targets for GBM treatment.

### Mechanosensitive ion channels: a therapeutic target in high-grade gliomas?

The key role of ion channels in GBM migration, progression and invasion, along with the upregulated expression of these ion channels in brain tumours, makes them promising targets to block. There are various antagonists available for TRP channels.^106^ For example, Ethyl-1-(4-(2,3,3-trichloroacrylamide)phenyl)-5-(trifluoromethyl)-1H-pyrazole-4-carboxylate (Pyr3), a selective TRPC3 channel antagonist, decreases LN229 glioma and U87-MG cell proliferation, migration and invasion.^107^ In a LN229-Luc2 mouse xenograft model, Pyr3 administration alongside TMZ inhibited GBM tumour growth.^107^ There are no specific antagonists for Piezo ion channels. Although no specific antagonists for Piezo1 currently exist, its activity can be inhibited by gadolinium, ruthenium red (a cationic dye), and Grammostola mechanotoxin 4 (GsMTx4; a spider venom peptide^108^). However, these compounds are non-specific and also block other cation-permeable mechanosensitive ion channels, including TRP channels.^109^ Both Piezo1^110^ and TRP^57^ ion channels are expressed widely across various organs throughout the body and are not specific to gliomas, even if they are overexpressed in gliomas compared to the healthy brain.^59^^,^^66^, ^67^, ^68^ Therefore, ion channel-specific and localised antagonism would be necessary to avoid off-target effects. Yet, it has been demonstrated that gadolinium inhibited Piezo- and TRP-dependent tissue damage during asthma attacks safely in vivo.^111^ However, targeting ion channels selectively through minimally invasive methods for targeted mechanobiology alterations would be preferable in terms of specificity and selectivity.

### FUS to modulate mechanobiology: strategic activation of ion channels

Ultrasound imaging uses high-frequency ultrasound waves to produce real-time anatomical images through wave reflection and scattering. Distinct from the established use of ultrasound for diagnostic imaging, focused ultrasound (FUS) is an emerging non-invasive medical technique with a wide range of therapeutic applications. A key distinction between diagnostic ultrasound imaging and FUS lies in the acoustic energy transfer and the bioeffects produced. While diagnostic ultrasound operates within safety limits designed to image the tissue without biological impact,^112^ FUS employs controlled acoustic parameters to modify the tissue and achieve therapeutic effects, including eliciting mechanical, ablative and/or thermal effects, depending on the parameters used. In FUS, ultrasound waves can be highly focused mechanically or electronically to target beams of acoustic energy at specific points within the body without damaging surrounding tissue.^113^ FUS is an attractive candidate for localised mechanical probing as parameters are highly adaptable whereby acoustic intensity, pressure, pulse repetition frequency (PRF), pulse length, and treatment depth can be altered for different purposes. Clinical applications of high-intensity ultrasound are currently being explored, including the ablation of peripheral and CNS tumours.^114^ Additionally, high-intensity FUS is routinely applied to ablate specific brain regions associated with Parkinson's disease to alleviate essential tremor.^115^

## Direct FUS bioeffects: evidence to date

### FUS effects on Piezo1

The effects of FUS have been explored on many cell types. However, the main research focus concerning FUS is neuromodulation, where FUS is used to alter neuronal activity, partly by stimulating several ion channels in neurons.^116^^,^^117^ Importantly, Piezo1 knock-out in mice significantly reduces ultrasound-induced neuronal calcium responses, muscle movement, and electromyogram signals,^118^ highlighting a key role for Piezo1 in ultrasound-induced neuromodulation.

In addition to neuromodulation, FUS has also been shown to activate Piezo1 in non-neuronal healthy cell lines (embryonic kidney,^119^ osteoblastic^120^) and cancerous cell lines (pancreatic cancer^121^ and breast cancer^122^^,^^123^). However, this activation has not been explored in brain cancer cells.

Table 1 summarises the studies exploring the effects of FUS-derived acoustic radiation forces on mechanosensitive ion channel Piezo1 in a variety of cell types in culture and some in vivo models. Low-intensity pulsed ultrasound has also been shown to promote Piezo1-induced intracellular calcium in osteoblastic cells.^120^ The importance of ultrasound pulse length in stimulating Piezo1 channels has been detailed in a study by Liao et al. (2021), using non-cancerous Piezo1 knock-out human embryonic kidney cells.^119^ This study found that the optimal pulse length of sonification for Piezo1 activation in human embryonic kidney cells under constant shear stress of 50 dyne/cm^2^ is 20 ms. Knockdown of Piezo1 using siRNA decreased intracellular calcium influx upon ultrasonic stimulation, indicating that Piezo1 activation may be influenced by shear stress generated by FUS.^119^

**Table 1:** Bioeffects of FUS on Piezo1: a summary of findings and parameters.

	References	Cell type	Findings	FUS parameters^a^	Ultrasound exposure vessel	In vitro Ca^2+^ imaging (Y/N)
Piezo1 activation	Liao et al., 2021^119^	Embryonic kidney cell line (HEK293T)	The optimal pulse length of sonification for Piezo1 activation under constant shear stress of 50 dyne/cm^2^ was PL = 20 ms.	Fc = 33 MHz, DC = 20%, PL = 20 ms, T = 1 min	Cell culture dish	Y
	Zhang et al., 2021^120^	Osteoblastic cell line (MC3T3-E1)	Results suggest that low-intensity pulsed ultrasound promotes Piezo1-induced intracellular calcium.	Fc = 2.25 MHz, DC = 20%, PRF = 1 Hz, T = 3 min	Cell culture dish	Y
Piezo1-mediated apoptosis	Song et al., 2022^121^	Pancreatic cancer cell lines (AsPC1, BxPC3, PANC1, HPNE)	Ultrasound with microbubbles induces apoptosis of pancreatic cancer cells. Ultrasound and microbubble-induced Piezo1 activation mediate calcium influx.	Fc = 1 MHz, I = 1.0 W/cm^2^, P = 176 kPa, DC = 50%, T = 10 min	Cell culture dish	Y
	Tijore et al., 2025^122^	Triple-negative breast cancer cell line (MDA-MB-231)	Low-frequency ultrasound induces apoptosis of MDA-MB-231 cells via activation of the calpain-dependent mitochondrial pathway and is reliant upon calcium entry through Piezo1 channels.	Fc = 33 kHz, T = 2 h, DC = 50%	96 well plate	Y
	Singh et al., 2021^123^	Triple-negative breast cancer cell line (MDA-MB-231)	Piezo1 is required for ultrasound-induced apoptosis in MDA-MB-231 cells.	Fc = 33 kHz, T = 2 h, DC = 50%	96 well plate	N

a FUS parameters include: Fc, centre frequency of the transducer in MHz; I, intensity of the acoustic field in W/cm^2^; W, ultrasonic stimulation power in Watts (W); T, Time in seconds (s) or minutes (min) or hours (h); PL, Pulse length in ms; DC, Duty cycle (%); NP, Number of pulses; and PRF, Pulse repetition frequency in Hz.

As detailed previously and depicted in Fig. 1, sustained activation of the mechanosensitive ion channel Piezo1 has been shown to drive tumour proliferation and facilitate increased tumour stiffness. However, acute activation of these channels has also been demonstrated to induce cancer cell death.^121^, ^122^, ^123^ In a pancreatic cancer model, the knockdown of Piezo1 reduced the therapeutic effect of ultrasound and microbubbles in vivo, providing evidence that Piezo1 is required for FUS-induced apoptosis.^121^ Previous work showed that Piezo1 activation is necessary for low-frequency ultrasound-induced apoptosis of breast cancer cells MDA-MB-231.^122^^,^^123^ Activation of the calpain-dependent mitochondrial pathway through calcium entry via Piezo1 ion channels has been shown to prompt ultrasound-induced apoptosis of MDA-MB-231 cells.^107^ Piezo1 shows promise as a therapeutic target for FUS in both pancreatic cancer^121^ and triple-negative breast cancer.^122^^,^^123^ Therefore, this target should be explored further as a treatment option for these cancers, as well as others, including GBM. Piezo1 activation has not been explored in GBM cells, however, the effect of FUS on cancer cells, including GBM cells, has been studied and will be discussed below.

**Fig. 2:**
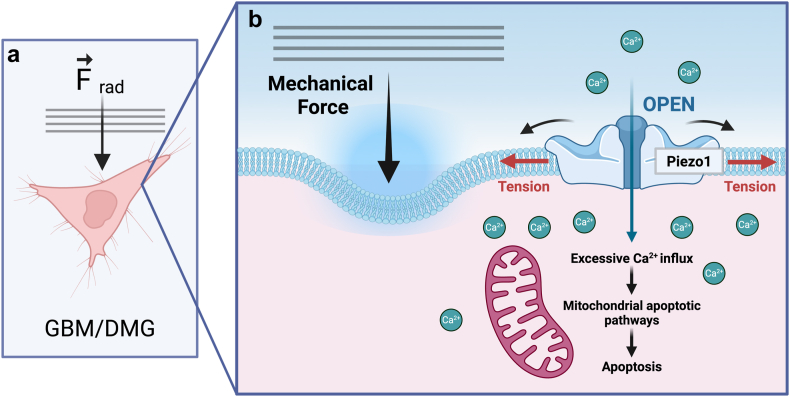
Proposed bioeffects of focused ultrasound (FUS) via activation of mechanosensitive ion channels in brain cancer cells. The right panel (b) shows a close-up of the proposed cellular events occurring in the left panel (a). (a) FUS generates acoustic radiation forces (F_rad_) that deform the membranes of brain tumour cells (e.g., glioblastoma [GBM] or diffuse midline glioma [DMG]). (b) This membrane deformation increases membrane tension, and this acute dynamic stimulation may activate mechanosensitive Piezo1 ion channels, triggering Ca^2+^ influx and promoting apoptosis via enhanced activation of mitochondrial apoptotic pathways. Created with BioRender.com.

Piezo1 is implicated in promoting tumour growth and stiffness, yet acute activation of these channels can also induce cell death in cancer cells in vitro^121^, ^122^, ^123^ and in vivo.^121^ These contrasting outcomes likely result from the distinct Piezo1 activation patterns; while prolonged activation may promote survival and proliferation,^67^^,^^105^ acute stimulation by FUS might trigger a cell death pathway^121^, ^122^, ^123^ (Fig. 2). Additionally, in vivo, cancer cells are exposed to various mechanical, biochemical, and environmental factors, including tissue stiffness, extracellular matrix interactions, and temporal variations in force application that are difficult to replicate in vitro.

### FUS effects on cancer cells: focus on GBM

FUS is being explored in a range of therapeutic applications within oncology due to its ability to elicit thermal and mechanical effects on tumour tissue, which can lead to tissue ablation due to the highly localised temperature increase.^124^ Additionally, applications within neuro-oncology include thermal ablation, drug release, sonodynamic therapy, immunomodulation, liquid biopsies and using FUS and microbubbles to enable transient blood-brain barrier (BBB) opening to enable improved drug delivery.^113^ FUS-mediated BBB opening has demonstrated safety in a mouse model of pontine glioma^125^ and has also shown feasibility and safety when combined with radiotherapy in a DMG mouse model.^126^ Furthermore, FUS-induced BBB opening effectively enhances chemotherapy drug delivery in mouse models, improving the efficacy of etoposide in a glioblastoma mouse model^127^ and panobinostat in a DMG mouse model.^128^ BBB opening safety has been evaluated in non-human primates.^129^ Several phase 1 clinical trials have also evaluated safety in BBB opening and delivery of chemotherapies in patients with high-grade gliomas,^130^, ^131^, ^132^ and larger-scale studies are recruiting.^133^ Ongoing and active clinical trials aim to evaluate the safety of BBB opening in humans across different tumour types, including DMG/DIPG.^133^ In addition to the delivery of chemotherapeutic agents, FUS has shown promise in pre-clinical models to facilitate delivery of a wide range of therapeutic agents of varying molecular sizes, including targeted gene therapy approaches such as siRNA^134^ and CRISPR-Cas9.^135^^,^^136^ Therefore, understanding the nuanced effects of FUS on brain tumour cells and how it may be integrated with surgical resection, radiotherapy, chemotherapy, and emerging gene-based therapies, is crucial for refining treatment strategies and optimising FUS parameters to achieve targeted and selective cancer cell death, while ensuring the safety and efficacy of therapeutic interventions, and without inducing detrimental downstream effects.

Several studies have explored the biological effects of FUS on glioblastoma and other cancerous cells in vitro (Table 2). For example, Landgraf et al. (2022), using GBM spheroids, demonstrated that FUS exposure reduced spheroid size, reduced cell metabolic activity (measured as cellular ATP levels), affected DNA repair, and increased DNA double-stranded breaks (measured as γH2A.X signal by FACS).^137^ GBM spheroids were formed using the U-87-MG cell line and exposed to FUS treatment at intensity of 2.95 W/cm^2^ and 5.9 W/cm^2^. An ATP metabolism assay in this study revealed that following FUS GBM spheroids showed a significant loss in ATP metabolism after FUS treatment at intensities of 5.9 W/cm^2^ and not 2.95 W/cm^2^, and this reduction in metabolic activity was seen at both 48 and 96 h after treatment. These studies highlight that FUS can elicit therapeutic effects on brain cancer cells in vitro. However, the mechanisms of action, including the effect on downstream cell signalling pathways following FUS exposure on elects on brain cells are still not well understood. Additionally, the role of Piezo1 in GBM cells in response to FUS remains uncharacterised.

**Table 2:** Bioeffects of FUS on GBM and other cancerous cells: a summary of findings and parameters.

References	Cell type	Findings	FUS Parameters^a^	Ultrasound exposure vessel	Radiation (Y/N)	In vitro Ca^2+^ imaging (Y/N)
Song et al., 2022^121^	Pancreatic cancer cell lines (AsPC1, BxPC3, PANC1, HPNE)	Ultrasound with microbubbles induces apoptosis of pancreatic cancer cells. Ultrasound and microbubble-induced Piezo1 activation mediate calcium influx.	Fc = 1 MHz, I = 1.0 W/cm^2^, P = 176 kPa, DC = 50%, T = 10 min	Cell culture dish	N	Y
Tijore et al., 2025^122^	Triple-negative breast cancer cell line (MDA-MB-231)	Low-frequency ultrasound induces apoptosis of MDA-MB-231 cells via activation of the calpain-dependent mitochondrial pathway and is reliant upon calcium entry through Piezo1 channels.	Fc = 33 kHz, T = 2 h, DC = 50%	96 well plate	N	Y
Singh et al., 2021^123^	Triple-negative breast cancer cell line (MDA-MB-231)	Piezo1 is required for ultrasound-induced apoptosis in MDA-MB-231 cells.	Fc = 33 kHz, T = 2 h, DC = 50%	96 well plate	N	N
Landgrad et al., 2022^137^	Glioblastoma (U-87) and prostate cancer (PC-3) cell lines	FUS reduced spheroid size and metabolic activity (cellular ATP) and increased DNA double-stranded breaks.	Fc = 1.1 MHz, I = 2.95 and 5.9 W/cm^2^, DC = 10% and 20%, PRF = 5 Hz, PL = 20 ms, T = 90 s	96 well plate	N	N
Rosenblatt et al., 2020^138^	Murine breast tumour (4T1), melanoma (B16), rat glioma (C6), and human breast tumour (MDA-MB-231) cell lines	Ca^2^^+^ transients during pulsed FUS generate reactive oxygen species (ROS) and cause DNA damage in tumour cells.	Fc = 1.1 MHz, P = 6 MPa, DC = 10%, PRF = 10 Hz, PL = 20 ms, NP = 300	500 μL centrifuge tube	N	Y
Zhang, X. et al., 2021^139^	Human head and neck cancer (FaDU), glioblastoma (T98G), and prostate cancer (PC-3) cell lines	FUS sensitises human cancer cells by enhancement of DNA damage.	Fc = 1.147 MHz and 1.467 MHz, T = 30 min	96 well plate	Y	N
Tutak et al., 2022^140^	Cancer stem cells and glioblastoma (U-87) cell lines	High doses of Voxtalisib+ low-intensity pulsed ultrasound inhibited mTOR signalling and decreased the viability in both cell groups.	I = 0.5 W/cm^2^, DC = 20%, PRF = 1 Hz, T = 30 s and 60 s	6 well plate	N	N

a FUS parameters include: Fc, centre frequency of the transducer in MHz; I, intensity of the acoustic field in W/cm^2^; W, ultrasonic stimulation power in Watts (W); T, Time in seconds (s) or minutes (min) or hours (h); PL, Pulse length in ms; DC, Duty cycle (%); NP, Number of pulses; and PRF, Pulse repetition frequency in Hz.

## Conclusions

As brain tumours advance, they often exhibit altered mechanobiology, which affects the treatment landscape. However, the changes in physiological properties and cellular function could be exploited to halt the disease progression and provide potential avenues for treatment. Mechanical hallmarks in brain cancer GBM have been explored. These include increased ECM stiffness,^43^^,^^44^ increased expression of mechanosensitive ion channels^59^^,^^66^, ^67^, ^68^ and altered expression of cell signalling pathways involved in apoptosis, cell proliferation, migration, and adhesion.^43^^,^^89^^,^^90^^,^^96^^,^^99^^,^^100^

Brain tumours, including GBM, require the development of novel therapeutics, and there is a clear rationale for exploring ion channels, such as TRP and Piezo, as therapeutic targets. Piezo1^66^, ^67^, ^68^ and certain TRP ion channels^59^^,^^60^ are upregulated in high-grade glioma samples compared to healthy and low-grade gliomas, suggesting that aggressive cancer cells are more sensitive to mechanical forces. However, further research is needed to understand the role of Piezo1 and TRP ion channels in glioma malignancy.

Exploiting this upregulation could be a way to selectively target the brain tumour using FUS. FUS can stimulate Piezo1 in various healthy^119^^,^^120^ and cancerous cell lines.^121^, ^122^, ^123^ However, this has not been shown in brain cancer cell lines or in vivo, so it is an area for future work to explore. The dual nature of Piezo1 signalling is pivotal: activation of Piezo1 and downstream pathways are involved in promoting stiffness and tumour progression,^36^^,^^41^^,^^55^, ^56^, ^57^^,^^75^^,^^82^^,^^85^^,^^86^^,^^93^^,^^102^^,^^103^ while acute dynamic stimulation via FUS can trigger mitochondrial apoptotic pathways and induce cancer cell death.^121^, ^122^, ^123^ The distinct activation patterns likely drive these contrasting outcomes, where prolonged activation promotes survival and acute dynamic activation via FUS triggers apoptosis. This complexity highlights the need for precise control of FUS parameters, particularly as its use expands in neuro-oncology.

Here, we discuss how targeting mechanosensitive ion channels and exploring the therapeutic potential of FUS may offer novel approaches to inhibit tumour progression and improve treatment outcomes for patients with brain tumours. FUS is being explored as a therapeutic strategy for diseases of the brain like brain tumours due to its ability to elicit mechanical, ablative and/or thermal effects.^141^ As presented in Table 1, Table 2, the impact of FUS on Piezo1 and GBM has been studied in vitro. However, only limited studies have explored the effect of FUS on brain cancer cells, and the effects of FUS on Piezo1 in brain cancer cells have not yet been investigated. Due to the overexpression of Piezo1 in GBM cells, which suggests Piezo1 as a prognostic marker and potential therapeutic target, exploration of FUS effects on Piezo1 in GBM cells presents a potential area for future research. FUS exposure could non-invasively mechanically activate Piezo1 in brain tumour cells (GBM and/or DMG), stimulating Ca^2+^ influx, which could initiate mitochondrial apoptotic pathways and promote cancer cell death (Fig. 2). Incorporating ion channel expression profiling,^142^ such as that of Piezo1, into tumour biopsy analysis or through emerging liquid biopsy approaches,^143^^,^^144^ could also represent a future opportunity to stratify patients with brain tumours most likely to benefit from FUS-based therapies. Targeting ion channels and signalling pathways disrupted by altered mechanobiology using remotely applied acoustic forces could be an effective non-invasive adjuvant therapy to reduce brain tumour progression. However, many of these mechanotransduction pathways can regulate pro-tumorigenic properties. Future work should focus on understanding how FUS affects brain tumour biology to ensure treatments reduce tumour progression rather than inadvertently promoting survival.

## Outstanding questions

A limited number of studies have explored the FUS bioeffect on cancer cells (Table 2), and several studies within those have explored the effect of FUS on brain cancer cell lines.^137^, ^138^, ^139^, ^140^ However, there is a growing interest in understanding the bioeffects of FUS on brain tumours, stemming from the increasing use of FUS within neuro-oncology research,^113^^,^^127^^,^^129^ indicating the rapid expansion of this research area. Future in vitro studies should make several considerations. For example, standardised reporting of ultrasound parameters, including focal dimensions and pressure gradients, would aid in comparisons across studies. The variability in the ultrasonic properties of vessels used during ultrasound exposure introduces potential inconsistencies due to differences in material properties and designs. Additionally, previous in vitro studies have not yet adequately modelled the in vivo ECM environment, which is crucial for understanding tumour mechanical response. The inclusion of the ECM, either by culturing cells with the addition of exogenous scaffolds, including ECM structural proteins, growth of cells in hydrogels that mimic varying tissue stiffness, or bioengineering strategies that allow cells to generate their own ECM in vitro,^145^ may offer a more accurate representation of tumour responses to FUS. Furthermore, a more comprehensive analysis of mechanotransduction across diverse brain tumour types is needed to refine FUS-based therapeutic strategies and ensure their efficacy across different tumour subtypes.

Microglia has been shown to highly express Piezo1.^146^ Piezo1 has also been shown to play a vital role in microglial migration and immune response.^146^ Therefore, non-specific targeting of Piezo1 in brain cells both within the brain TME and within healthy proximal brain tissue during FUS mechanical modulation could induce off-target effects, necessitating the characterisation of the effect of FUS on all brain cell types. Determining the overall positive effects of FUS on brain tumours and the effects on other brain cells will help elucidate whether the benefits outweigh the risks of off-target effects. Equally, FUS stimulation of glial cells, such as microglia, could potentiate the therapeutic effect of FUS via immunomodulation. We refer readers to a recent comprehensive review detailing FUS bioeffects on microglial activity in both healthy and neurological disease contexts.^147^ Future work could explore the effect of FUS on proximal neighbouring healthy brain cells in vivo using an animal model of GBM and/or DMG.

## Contributors

LG: Conceptualisation, Writing – original draft, Writing – review and editing. CPP: Conceptualisation, Writing – original draft, Writing – review and editing. JR: Conceptualisation, Funding acquisition, Resources, Supervision, Writing – review and editing. ANP: Conceptualisation, Funding acquisition, Resources, Supervision, Writing – review and editing. All authors have read and approved the final version of the manuscript.

## Declaration of interests

All authors declare no competing interests.

## References

[1] Louis D.N., Perry A., Wesseling P. (2021). The 2021 WHO classification of tumors of the central nervous system: a summary. Neuro Oncol.

[2] Stupp R., Hegi M.E., Mason W.P. (2009). Effects of radiotherapy with concomitant and adjuvant temozolomide versus radiotherapy alone on survival in glioblastoma in a randomised phase III study: 5-year analysis of the EORTC-NCIC trial. Lancet Oncol.

[3] Koshy M., Villano J.L., Dolecek T.A. (2012). Improved survival time trends for glioblastoma using the SEER 17 population-based registries. J Neurooncol.

[4] Kim H.J., Park J.W., Lee J.H. (2021). Genetic architectures and cell-of-origin in glioblastoma. Front Oncol.

[5] Eisenbarth D., Wang Y.A. (2023). Glioblastoma heterogeneity at single cell resolution. Oncogene.

[6] Drumm M.R., Dixit K.S., Grimm S. (2020). Extensive brainstem infiltration, not mass effect, is a common feature of end-stage cerebral glioblastomas. Neuro Oncol.

[7] Chen W., Wang Y., Zhao B. (2021). Optimal therapies for recurrent glioblastoma: a bayesian network meta-analysis. Front Oncol.

[8] Venkataramani V., Yang Y., Schubert M.C. (2022). Glioblastoma hijacks neuronal mechanisms for brain invasion. Cell.

[9] Monje M., Mitra S.S., Freret M.E. (2011). Hedgehog-responsive candidate cell of origin for diffuse intrinsic pontine glioma. Proc Natl Acad Sci U S A.

[10] Filbin M.G., Tirosh I., Hovestadt V. (2018). Developmental and oncogenic programs in H3K27M gliomas dissected by single-cell RNA-seq. Science.

[11] Nagaraja S., Vitanza N.A., Woo P.J. (2017). Transcriptional dependencies in diffuse intrinsic pontine glioma. Cancer Cell.

[12] Al Sharie S., Abu Laban D., Al-Hussaini M. (2023). Decoding diffuse midline gliomas: a comprehensive review of pathogenesis, diagnosis and treatment. Cancers (Basel).

[13] Persson M.L., Douglas A.M., Alvaro F. (2022). The intrinsic and microenvironmental features of diffuse midline glioma: implications for the development of effective immunotherapeutic treatment strategies. Neuro Oncol.

[14] Jang S.W., Song S.W., Kim Y.H. (2022). Clinical features and prognosis of diffuse midline glioma: a series of 24 cases. Brain Tumor Res Treat.

[15] Diaz M.J., Kwak S.H., Root K.T. (2022). Current approaches to craniopharyngioma management. Front Biosci (Landmark Ed).

[16] Seano G., Nia H.T., Emblem K.E. (2019). Solid stress in brain tumours causes neuronal loss and neurological dysfunction and can be reversed by lithium. Nat Biomed Eng.

[17] Jain R.K., Martin J.D., Stylianopoulos T. (2014). The role of mechanical forces in tumor growth and therapy. Annu Rev Biomed Eng.

[18] Mierke C.T. (2024). Extracellular matrix cues regulate mechanosensing and mechanotransduction of cancer cells. Cells.

[19] Bera K., Kiepas A., Godet I. (2022). Extracellular fluid viscosity enhances cell migration and cancer dissemination. Nature.

[20] Martino F., Perestrelo A.R., Vinarský V., Pagliari S., Forte G. (2018). Cellular mechanotransduction: from tension to function. Front Physiol.

[21] Kechagia J.Z., Ivaska J., Roca-Cusachs P. (2019). Integrins as biomechanical sensors of the microenvironment. Nat Rev Mol Cell Biol.

[22] Kanchanawong P., Calderwood D.A. (2023). Organization, dynamics and mechanoregulation of integrin-mediated cell–ECM adhesions. Nat Rev Mol Cell Biol.

[23] Hoffman B.D., Yap A.S. (2015). Towards a dynamic understanding of cadherin-based mechanobiology. Trends Cell Biol.

[24] Budnar S., Yap A.S. (2013). A mechanobiological perspective on cadherins and the actin-myosin cytoskeleton. F1000Prime Rep.

[25] Elias A.F., Lin B.C., Piggott B.J. (2023). Ion channels in gliomas—from molecular basis to treatment. Int J Mol Sci.

[26] Karska J., Kowalski S., Saczko J., Moisescu M.G., Kulbacka J. (2023). Mechanosensitive ion channels and their role in cancer cells. Membranes (Basel).

[27] Ranade S.S., Syeda R., Patapoutian A. (2015). Mechanically activated ion channels. Neuron.

[28] Gu Y., Gu C. (2014). Physiological and pathological functions of mechanosensitive ion channels. Mol Neurobiol.

[29] Jin P., Jan L.Y., Jan Y.N. (2020). Mechanosensitive ion channels: structural features relevant to mechanotransduction mechanisms. Annu Rev Neurosci.

[30] Winkler J., Abisoye-Ogunniyan A., Metcalf K.J., Werb Z. (2020). Concepts of extracellular matrix remodelling in tumour progression and metastasis. Nat Commun.

[31] Huang J., Zhang L., Wan D. (2021). Extracellular matrix and its therapeutic potential for cancer treatment. Signal Transduct Target Ther.

[32] Karamanos N.K., Theocharis A.D., Piperigkou Z. (2021). A guide to the composition and functions of the extracellular matrix. FEBS J.

[33] Pang X., He X., Qiu Z. (2023). Targeting integrin pathways: mechanisms and advances in therapy. Signal Transduct Target Ther.

[34] Mezu-Ndubuisi O.J., Maheshwari A. (2021). The role of integrins in inflammation and angiogenesis. Pediatr Res.

[35] McHugh B.J., Buttery R., Lad Y., Banks S., Haslett C., Sethi T. (2010). Integrin activation by Fam38A uses a novel mechanism of R-Ras targeting to the endoplasmic reticulum. J Cell Sci.

[36] Hiscox L.V., Johnson C.L., McGarry M.D.J. (2020). Mechanical property alterations across the cerebral cortex due to Alzheimer's disease. Brain Commun.

[37] Svensson S.F., De Arcos J., Darwish O.I. (2021). Robustness of MR elastography in the healthy brain: repeatability, reliability, and effect of different reconstruction methods. J Magn Reson Imaging.

[38] Babu P.K.V., Radmacher M. (2019). Mechanics of brain tissues studied by atomic force microscopy: a perspective. Front Neurosci.

[39] Fuhs T., Flachmeyer B., Krueger M., Blietz A., Härtig W., Michalski D. (2023). Combining atomic force microscopy and fluorescence-based techniques to explore mechanical properties of naive and ischemia-affected brain regions in mice. Sci Rep.

[40] Discher D.E., Janmey P., Wang Y.L. (2005). Tissue cells feel and respond to the stiffness of their substrate. Science.

[41] Hall C.M., Moeendarbary E., Sheridan G.K. (2021). Mechanobiology of the brain in ageing and Alzheimer's disease. Eur J Neurosci.

[42] Elosegui-Artola A. (2021). The extracellular matrix viscoelasticity as a regulator of cell and tissue dynamics. Curr Opin Cell Biol.

[43] Miroshnikova Y.A., Mouw J.K., Barnes J.M. (2016). Tissue mechanics promote IDH1-dependent HIF1α-tenascin C feedback to regulate glioblastoma aggression. Nat Cell Biol.

[44] Sohrabi A., Lefebvre A.E.Y.T., Harrison M.J. (2023). Microenvironmental stiffness induces metabolic reprogramming in glioblastoma. Cell Rep.

[45] Streitberger K.J., Reiss-Zimmermann M., Freimann F.B. (2014). High-resolution mechanical imaging of glioblastoma by multifrequency magnetic resonance elastography. PLoS One.

[46] Pepin K.M., McGee K.P., Arani A. (2018). MR elastography analysis of glioma stiffness and IDH1-mutation status. Am J Neuroradiol.

[47] Fløgstad Svensson S., Fuster-Garcia E., Latysheva A. (2022). Decreased tissue stiffness in glioblastoma by MR elastography is associated with increased cerebral blood flow. Eur J Radiol.

[48] Umesh V., Rape A.D., Ulrich T.A., Kumar S. (2014). Microenvironmental stiffness enhances glioma cell proliferation by stimulating epidermal growth factor receptor signaling. PLoS One.

[49] Ulrich T.A., De Juan Pardo E.M., Kumar S. (2009). The mechanical rigidity of the extracellular matrix regulates the structure, motility, and proliferation of glioma cells. Cancer Res.

[50] DuChez B.J., Doyle A.D., Dimitriadis E.K., Yamada K.M. (2019). Durotaxis by human cancer cells. Biophys J.

[51] Isomursu A., Park K.Y., Hou J. (2022). Directed cell migration towards softer environments. Nat Mater.

[52] Yuan Z., Li Y., Zhang S. (2023). Extracellular matrix remodeling in tumor progression and immune escape: from mechanisms to treatments. Mol Cancer.

[53] de Visser K.E., Joyce J.A. (2023). The evolving tumor microenvironment: from cancer initiation to metastatic outgrowth. Cancer Cell.

[54] Popova N.V., Jücker M. (2022). The functional role of extracellular matrix proteins in cancer. Cancers (Basel).

[55] Schregel K., Nazari N., Nowicki M.O. (2018). Characterization of glioblastoma in an orthotopic mouse model with magnetic resonance elastography. NMR Biomed.

[56] Streitberger K.J., Lilaj L., Schrank F. (2020). How tissue fluidity influences brain tumor progression. Proc Natl Acad Sci U S A.

[57] Samanta A., Hughes T.E.T., Moiseenkova-Bell V.Y. (2018). Transient receptor potential (TRP) channels. Subcell Biochem.

[58] Cox C.D., Poole K., Martinac B. (2024). Re-evaluating TRP channel mechanosensitivity. Trends Biochem Sci.

[59] Alptekin M., Eroglu S., Tutar E. (2015). Gene expressions of TRP channels in glioblastoma multiforme and relation with survival. Tumour Biol.

[60] Leng T.D., Li M.H., Shen J.F. (2015). Suppression of TRPM7 inhibits proliferation, migration, and invasion of malignant human glioma cells. CNS Neurosci Ther.

[61] Wong R., Gong H., Alanazi R. (2020). Inhibition of TRPM7 with waixenicin A reduces glioblastoma cellular functions. Cell Calcium.

[62] Coste B., Mathur J., Schmidt M. (2010). Piezo1 and Piezo2 are essential components of distinct mechanically activated cation channels. Science.

[63] Vasileva V.Y., Lysikova D.V., Sudarikova A.V. (2024). Functional characterization of native Piezo1 as calcium and magnesium influx pathway in human myeloid leukemia cells. J Cell Physiol.

[64] Pathak M.M., Nourse J.L., Tran T. (2014). Stretch-activated ion channel Piezo1 directs lineage choice in human neural stem cells. Proc Natl Acad Sci U S A.

[65] Hong R., Yang D., Jing Y., Chen S., Tian H., Yang Y. (2023). PIEZO1-related physiological and pathological processes in CNS: focus on the gliomas. Cancers (Basel).

[66] Qu S., Li S., Hu Z. (2020). Upregulation of Piezo1 is a novel prognostic indicator in glioma patients. Cancer Manag Res.

[67] Chen X., Wanggou S., Bodalia A. (2018). A feedforward mechanism mediated by mechanosensitive ion channel PIEZO1 and tissue mechanics promotes glioma aggression. Neuron.

[68] Zhou W., Liu X., van Wijnbergen J.W.M. (2020). Identification of PIEZO1 as a potential prognostic marker in gliomas. Sci Rep.

[69] Wang L., Zhou H., Zhang M. (2019). Structure and mechanogating of the mammalian tactile channel PIEZO2. Nature.

[70] Servin-Vences M.R., Lam R.M., Koolen A. (2023). PIEZO2 in somatosensory neurons controls gastrointestinal transit. Cell.

[71] Woo S.H., Ranade S., Weyer A.D. (2014). Piezo2 is required for Merkel-cell mechanotransduction. Nature.

[72] Chang W., Gu J.G. (2020). Role of microtubules in Piezo2 mechanotransduction of mouse Merkel cells. J Neurophysiol.

[73] Yang H., Liu C., Zhou R.M. (2016). Piezo2 protein: a novel regulator of tumor angiogenesis and hyperpermeability. Oncotarget.

[74] Chen X., Momin A., Wanggou S. (2023). Mechanosensitive brain tumor cells construct blood-tumor barrier to mask chemosensitivity. Neuron.

[75] Pardo-Pastor C., Rubio-Moscardo F., Vogel-González M. (2018). Piezo2 channel regulates RhoA and actin cytoskeleton to promote cell mechanobiological responses. Proc Natl Acad Sci U S A.

[76] Valiente M., Obenauf A.C., Jin X. (2014). Serpins promote cancer cell survival and vascular co-option in brain metastasis. Cell.

[77] Elosegui-Artola A., Oria R., Chen Y. (2016). Mechanical regulation of a molecular clutch defines force transmission and transduction in response to matrix rigidity. Nat Cell Biol.

[78] Shah N., Mohammad A.S., Saralkar P. (2018). Investigational chemotherapy and novel pharmacokinetic mechanisms for the treatment of breast cancer brain metastases. Pharmacol Res.

[79] Baroja I., Kyriakidis N.C., Halder G., Moya I.M. (2024). Expected and unexpected effects after systemic inhibition of Hippo transcriptional output in cancer. Nat Commun.

[80] Piccolo S., Panciera T., Contessotto P., Cordenonsi M. (2023). YAP/TAZ as master regulators in cancer: modulation, function and therapeutic approaches. Nat Cancer.

[81] Ortega Á., Vera I., Diaz M.P. (2022). The YAP/TAZ signaling pathway in the tumor microenvironment and carcinogenesis: current knowledge and therapeutic promises. Int J Mol Sci.

[82] Zhang X., Abdelrahman A., Vollmar B., Zechner D. (2018). The ambivalent function of YAP in apoptosis and cancer. Int J Mol Sci.

[83] Dupont S., Morsut L., Aragona M. (2011). Role of YAP/TAZ in mechanotransduction. Nature.

[84] Elosegui-Artola A., Andreu I., Beedle A.E.M. (2017). Force triggers YAP nuclear entry by regulating transport across nuclear pores. Cell.

[85] Zhu B., Qian W., Han C., Bai T., Hou X. (2021). Piezo 1 activation facilitates cholangiocarcinoma metastasis via Hippo/YAP signaling axis. Mol Ther Nucleic Acids.

[86] Pardo-Pastor C., Rosenblatt J. (2023). Piezo1 activates noncanonical EGFR endocytosis and signaling. Sci Adv.

[87] Hasegawa K., Fujii S., Matsumoto S., Tajiri Y., Kikuchi A., Kiyoshima T. (2021). YAP signaling induces PIEZO1 to promote oral squamous cell carcinoma cell proliferation. J Pathol.

[88] Han Y. (2019). Analysis of the role of the Hippo pathway in cancer. J Transl Med.

[89] Vigneswaran K., Boyd N.H., Oh S.Y. (2021). YAP/TAZ transcriptional coactivators create therapeutic vulnerability to verteporfin in EGFR-mutant glioblastoma. Clin Cancer Res.

[90] Zhou J., Yi Q., Tang L. (2019). The roles of nuclear focal adhesion kinase (FAK) on Cancer: a focused review. J Exp Clin Cancer Res.

[91] Pan Y., Shi L.Z., Yoon C.W. (2022). Mechanosensor Piezo1 mediates bimodal patterns of intracellular calcium and FAK signaling. EMBO J.

[92] Tan X., Yan Y., Song B., Zhu S., Mei Q., Wu K. (2023). Focal adhesion kinase: from biological functions to therapeutic strategies. Exp Hematol Oncol.

[93] Alza L., Nàger M., Visa A., Cantí C., Herreros J. (2020). FAK inhibition induces glioblastoma cell senescence-like state through p62 and p27. Cancers (Basel).

[94] Schmitt C.A., Wang B., Demaria M. (2022). Senescence and cancer — role and therapeutic opportunities. Nat Rev Clin Oncol.

[95] Brown N.F., Williams M., Arkenau H.T. (2018). A study of the focal adhesion kinase inhibitor GSK2256098 in patients with recurrent glioblastoma with evaluation of tumor penetration of [11C]GSK2256098. Neuro Oncol.

[96] Rascio F., Spadaccino F., Rocchetti M.T. (2021). The pathogenic role of PI3K/AKT pathway in cancer onset and drug resistance: an updated review. Cancers (Basel).

[97] Eckerdt F.D., Bell J.B., Gonzalez C. (2020). Combined PI3Kα-mTOR targeting of glioma stem cells. Sci Rep.

[98] Zhang S., Cao S., Gong M. (2022). Mechanically activated ion channel Piezo1 contributes to melanoma malignant progression through AKT/mTOR signaling. Cancer Biol Ther.

[99] Saadeh F.S., Mahfouz R., Assi H.I. (2018). Egfr as a clinical marker in glioblastomas and other gliomas. Int J Biol Markers.

[100] Xu H., Zong H., Ma C. (2017). Epidermal growth factor receptor in glioblastoma. Oncol Lett.

[101] Li X., Wu C., Chen N. (2016). PI3K/Akt/mTOR signaling pathway and targeted therapy for glioblastoma. Oncotarget.

[102] Pelloski C.E., Ballman K.V., Furth A.F. (2007). Epidermal growth factor receptor variant III status defines clinically distinct subtypes of glioblastoma. J Clin Oncol.

[103] An Z., Aksoy O., Zheng T., Fan Q.W., Weiss W.A. (2018). Epidermal growth factor receptor and EGFRvIII in glioblastoma: signaling pathways and targeted therapies. Oncogene.

[104] Hu C., Leche C.A., Kiyatkin A. (2022). Glioblastoma mutations alter EGFR dimer structure to prevent ligand bias. Nature.

[105] Chuang H.H., Zhen Y.Y., Tsai Y.C. (2022). FAK in cancer: from mechanisms to therapeutic strategies. Int J Mol Sci.

[106] Koivisto A.P., Belvisi M.G., Gaudet R., Szallasi A. (2022). Advances in TRP channel drug discovery: from target validation to clinical studies. Nat Rev Drug Discov.

[107] Chang H.H., Cheng Y.C., Tsai W.C., Tsao M.J., Chen Y. (2018). Pyr3 induces apoptosis and inhibits migration in human glioblastoma cells. Cell Physiol Biochem.

[108] Thien N.D., Hai-Nam N., Anh D.T., Baecker D. (2024). Piezo1 and its inhibitors: overview and perspectives. Eur J Med Chem.

[109] Pumroy R.A., De Jesús-Pérez J.J., Protopopova A.D. (2024). Molecular details of ruthenium red pore block in TRPV channels. EMBO Rep.

[110] Wu J., Lewis A.H., Grandl J. (2017). Touch, Tension, and transduction – the function and regulation of piezo ion channels. Trends Biochem Sci.

[111] Bagley D.C., Russell T., Ortiz-Zapater E. (2024). Bronchoconstriction damages airway epithelia by crowding-induced excess cell extrusion. Science.

[112] Şen T., Tüfekçioğlu O., Koza Y. (2015). Mechanical index. Anadolu Kardiyol Derg.

[113] Meng Y., Hynynen K., Lipsman N. (2021). Applications of focused ultrasound in the brain: from thermoablation to drug delivery. Nat Rev Neurol.

[114] De Maio A., Alfieri G., Mattone M., Ghanouni P., Napoli A. (2024). High-intensity focused ultrasound surgery for tumor ablation: a review of current applications. Radiol Imaging Cancer.

[115] Baek H., Lockwood D., Mason E.J. (2022). Clinical intervention using focused ultrasound (FUS) stimulation of the brain in diverse neurological disorders. Front Neurol.

[116] Chu Y.C., Lim J., Chien A., Chen C.C., Wang J.L. (2022). Activation of mechanosensitive ion channels by ultrasound. Ultrasound Med Biol.

[117] Yoo S., Mittelstein D.R., Hurt R.C., Lacroix J., Shapiro M.G. (2022). Focused ultrasound excites cortical neurons via mechanosensitive calcium accumulation and ion channel amplification. Nat Commun.

[118] Zhu J., Xian Q., Hou X. (2023). The mechanosensitive ion channel Piezo1 contributes to ultrasound neuromodulation. Proc Natl Acad Sci U S A.

[119] Liao D., Hsiao M.Y., Xiang G., Zhong P. (2021). Optimal pulse length of insonification for Piezo1 activation and intracellular calcium response. Sci Rep.

[120] Zhang G., Li X., Wu L., Qin Y.X. (2021). Piezo1 channel activation in response to mechanobiological acoustic radiation force in osteoblastic cells. Bone Res.

[121] Song Y., Chen J., Zhang C. (2022). Mechanosensitive channel Piezo1 induces cell apoptosis in pancreatic cancer by ultrasound with microbubbles. iScience.

[122] Tijore A., Margadant F., Dwivedi N. (2025). Ultrasound-mediated mechanical forces activate selective tumor cell apoptosis. Bioeng Transl Med.

[123] Singh A., Tijore A., Margadant F. (2021). Enhanced tumor cell killing by ultrasound after microtubule depolymerization. Bioeng Transl Med.

[124] Diaz-Alejo J.F., Gomez I.G., Earl J. (2022). Ultrasounds in cancer therapy: a summary of their use and unexplored potential. Oncol Rev.

[125] Englander Z.K., Wei H.J., Pouliopoulos A.N. (2021). Focused ultrasound mediated blood–brain barrier opening is safe and feasible in a murine pontine glioma model. Sci Rep.

[126] Tazhibi M., McQuillan N., Wei H.J. (2024). Focused ultrasound-mediated blood–brain barrier opening is safe and feasible with moderately hypofractionated radiotherapy for brainstem diffuse midline glioma. J Transl Med.

[127] Wei H.J., Upadhyayula P.S., Pouliopoulos A.N. (2021). Focused ultrasound-mediated blood-brain barrier opening increases delivery and efficacy of etoposide for glioblastoma treatment. Int J Radiat Oncol Biol Phys.

[128] Martinez P., Nault G., Steiner J. (2023). MRI-guided focused ultrasound blood-brain barrier opening increases drug delivery and efficacy in a diffuse midline glioma mouse model. Neurooncol Adv.

[129] Pouliopoulos A.N., Kwon N., Jensen G. (2021). Safety evaluation of a clinical focused ultrasound system for neuronavigation guided blood-brain barrier opening in non-human primates. Sci Rep.

[130] Farzad Maroufi S., Sadegh Fallahi M., Parmis Maroufi S., Sheehan J.P. (2024). Focused ultrasound blood-brain barrier disruption in high-grade gliomas: scoping review of clinical studies. J Clin Neurosci.

[131] Sonabend A.M., Gould A., Amidei C. (2023). Repeated blood–brain barrier opening with an implantable ultrasound device for delivery of albumin-bound paclitaxel in patients with recurrent glioblastoma: a phase 1 trial. Lancet Oncol.

[132] Zhu H., Allwin C., Bassous M.G., Pouliopoulos A.N. (2024). Focused ultrasound-mediated enhancement of blood–brain barrier permeability for brain tumor treatment: a systematic review of clinical trials. J Neurooncol.

[133] Lu V.M., Niazi T.N. (2025). Clinical trials of focused ultrasound for brain tumors. Cancers (Basel).

[134] Guo Y., Lee H., Fang Z. (2021). Single-cell analysis reveals effective siRNA delivery in brain tumors with microbubble-enhanced ultrasound and cationic nanoparticles. Sci Adv.

[135] Lao Y.H., Ji R., Zhou J.K. (2023). Focused ultrasound–mediated brain genome editing. Proc Natl Acad Sci U S A.

[136] Gumusgoz E., Kasiri S., Youssef I. (2025). Focused ultrasound widely broadens AAV-delivered Cas9 distribution and activity. Gene Ther.

[137] Landgraf L., Kozlowski A., Zhang X. (2022). Focused ultrasound treatment of a spheroid in vitro tumour model. Cells.

[138] Rosenblatt R.B., Frank J.A., Burks S.R. (2020). Cytosolic Ca2+ transients during pulsed focused ultrasound generate reactive oxygen species and cause DNA damage in tumor cells. Theranostics.

[139] Zhang X., Bobeica M., Unger M. (2021). Focused ultrasound radiosensitizes human cancer cells by enhancement of DNA damage. Strahlenther Onkol.

[140] Tutak I., Ozdil B., Uysal A. (2022). Voxtalisib and low intensity pulsed ultrasound combinatorial effect on glioblastoma multiforme cancer stem cells via PI3K/AKT/mTOR. Pathol Res Pract.

[141] Nwafor D.C., Obiri-Yeboah D., Fazad F., Blanks W., Mut M. (2024). Focused ultrasound as a treatment modality for gliomas. Front Neurol.

[142] Wang R., Gurguis C.I., Gu W. (2015). Ion channel gene expression predicts survival in glioma patients. Sci Rep.

[143] Zhu L., Cheng G., Ye D. (2018). Focused ultrasound-enabled brain tumor liquid biopsy. Sci Rep.

[144] Saenz-Antoñanzas A., Auzmendi-Iriarte J., Carrasco-Garcia E. (2019). Liquid biopsy in glioblastoma: opportunities, applications and challenges. Cancers (Basel).

[145] Urciuolo F., Imparato G., Netti P.A. (2023). In vitro strategies for mimicking dynamic cell–ECM reciprocity in 3D culture models. Front Bioeng Biotechnol.

[146] Zhu T., Guo J., Wu Y. (2023). The mechanosensitive ion channel Piezo1 modulates the migration and immune response of microglia. iScience.

[147] Grewal S., Gonçalves de Andrade E., Kofoed R.H. (2023). Using focused ultrasound to modulate microglial structure and function. Front Cell Neurosci.

